# Therapeutic Potential of Mesenchymal Stem Cells in the Treatment of Ocular Graft-Versus-Host Disease

**DOI:** 10.3390/ijms232113254

**Published:** 2022-10-31

**Authors:** Carl Randall Harrell, Valentin Djonov, Vladislav Volarevic

**Affiliations:** 1 Regenerative Processing Plant, LLC, 34176 US Highway 19 N, Palm Harbor, FL 34684, USA; 2 Institute of Anatomy, University of Bern, Baltzerstrasse 2, 3012 Bern, Switzerland; 3 Department of Genetics, Faculty of Medical Sciences, University of Kragujevac, 69 Svetozar Markovic Street, 34000 Kragujevac, Serbia; 4 Department of Microbiology and Immunology, Faculty of Medical Sciences, University of Kragujevac, 69 Svetozar Markovic Street, 34000 Kragujevac, Serbia

**Keywords:** mesenchymal stem cells, exosomes, therapy, immunosuppression, ocular graft-versus-host disease

## Abstract

Ocular GVHD (oGVHD), manifested by severe injury of corneal epithelial cells, meibomian and lacrimal glands’ dysfunction, is a serious complication of systemic GVHD which develops as a consequence of donor T and natural killer cell-driven inflammation in the eyes of patients who received allogeneic hematopoietic stem cell transplantation. Mesenchymal stem cells (MSC) are, due to their enormous differentiation potential and immunosuppressive characteristics, considered as a potentially new remedy in ophthalmology. MSC differentiate in corneal epithelial cells, suppress eye inflammation, and restore meibomian and lacrimal glands’ function in oGVHD patients. MSC-sourced exosomes (MSC-Exos) are extracellular vesicles that contain MSC-derived growth factors and immunoregulatory proteins. Due to the lipid membrane and nano-sized dimension, MSC-Exos easily by-pass all biological barriers in the eyes and deliver their cargo directly in injured corneal epithelial cells and eye-infiltrated leukocytes, modulating their viability and function. As cell-free agents, MSC-Exos address all safety issues related to the transplantation of their parental cells, including the risk of unwanted differentiation and aggravation of intraocular inflammation. In this review article, we summarized current knowledge about molecular mechanisms which are responsible for beneficial effects of MSC and MSC-Exos in the therapy of inflammatory eye diseases, emphasizing their therapeutic potential in the treatment of oGVHD.

## 1. Introduction

Graft-versus-host disease (GVHD) is a severe, systemic disorder which develops as a serious and life-threatening complication of allogeneic hematopoietic stem cell (HSC) transplantation [1]. GVHD predominantly occurs in the skin, gastrointestinal tract, liver, oral mucosa and in the eyes of patients as a result of immune dysregulation and inflammation-mediated tissue destruction that lead to fibrosis and organ dysfunction [1]. The acute form of GVHD (aGVHD) is diagnosed if pathological events developed within 100 days after HSC transplantation, while the diagnosis of chronic GVHD disease (cGVHD) is made when GVHD-related clinical signs were observed 100 days after HSC transplantation [2,3].

**Figure 1:**
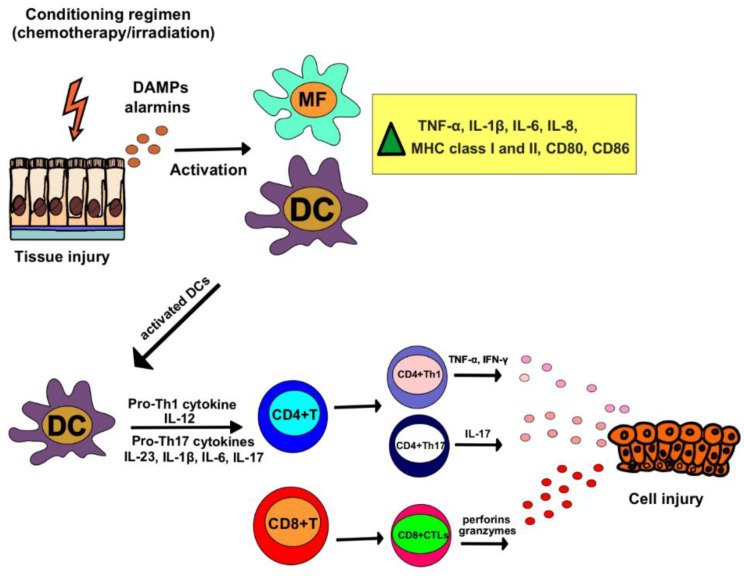
Pathophysiology of oGVHD. Chemotherapy and irradiation, which are used as “precondition treatment” prior to allogeneic HSC transplantation, induce release of damage-associated molecular patterns (DAMPs) and alarmins from injured parenchymal cells, which, in turn, activate tissue resident macrophages and dendritic cells (DCs) to produce large amount of inflammatory cytokines (tumor necrosis factor alpha (TNF-α), IL-1 beta (IL-1β), IL-6, IL-8) and to increase expression of major histocompatibility complex (MHC) and co-stimulatory molecules (CD80, CD86) on their membranes. Activated DCs capture antigens from damaged cells and bring them into the regional lymph nodes to activate donor CD4+ T helper and CD8+ cytotoxic T cells. DC-derived IL-12 is responsible for the development of Th1 cells, while DC-sourced IL-1β, IL-6 and IL-23 induce generation of effector Th17 cells. In addition to CD4+ T helper cells, perforin and granzyme B-producing CD8+cytotoxic T cells (CTLs) are also activated by DCs. Through the production of inflammatory cytokines and cytotoxic molecules, CD4+ and CD8+ T cells induce tissue injury in the eyes of oGVHD patients.

Immunoablative chemotherapy and irradiation, which are used as the “precondition treatment” prior to allogeneic HSC transplantation, are considered as the main risk factors for the development of aGVHD (Figure 1) [2,4]. Damage-associated molecular patterns (DAMPs) and alarmins (heat-shock proteins, interleukin (IL)-33), released from chemo/radiotherapy-induced injured parenchymal cells, activate tissue resident macrophages to produce large amount of inflammatory cytokines (tumor necrosis factor alpha (TNF-α), IL-1 beta (IL-1β), IL-6, IL-8) which induce enhanced expression of the major histocompatibility complex (MHC) and co-stimulatory molecules (CD80, CD86) on the membrane of professional antigen-presenting dendritic cells (DCs) [4]. Activated DCs capture antigens from damaged cells and bring them into the regional lymph nodes to activate donor CD4+ T helper and CD8+ cytotoxic T cells [4]. Inflammatory, IFN-γ-producing CD4+Th1 and IL-17-producing CD4+Th17 lymphocytes are considered as the main effector cells in the pathogenesis of aGVHD [2,4]. DC-derived IL-12 is crucially responsible for the development of Th1 cells, while DC-sourced IL-1β, IL-6 and IL-23 induce the generation of effector Th17 cells [4]. In addition to CD4+ T helper cells, perforin and granzyme B-producing CD8+cytotoxic T cells (CTLs) and natural killer (NK) cells are also involved in the tissue destruction during the progression of aGVHD [2,4]. Long-term tissue destruction driven by Th1, Th17, CTLs, and NK cells results in the development of cGVHD [3,4]. IFN-γ and IL-17 derived from Th1 and Th17 cells activate tissue resident macrophages and circulating neutrophils which, in turn, produce matrix metalloproteinases (MMPs), inflammatory cytokines, and pro-fibrotic transforming growth factor beta (TGF-β) resulting in fibrosis and organ dysfunction [3,4].

T-cell-recruiting chemokines (CXCL3, CXCL9, and CXCL11) and inflammatory cytokines (TNF-α, IL-1β, IL-6, IFN-γ) are highly elevated in the in the tears and at the ocular surface of patients suffering from GVHD [5]. Conjunctiva-associated mucosal tissue mimics systemic mucosal membranes of the lungs, intestines, and mouth, making it an ideal target for activated inflammatory cells [5,6]. Accordingly, although all components of the ocular surface (cornea, conjunctiva, eyelids, lacrimal glands, meibomian glands, and lacrimal drainage system) may be injured during the progression of ocular GVHD (oGVHD), keratoconjunctivitis sicca (dry eye disease (DED)) is the most frequent clinical sign of oGVHD [5,6,7]. It usually develops 6 to 12 months after HSC transplantation and is observed in the majority (up to 70%) of patients suffering from cGVHD [3,6]. Th1 and Th17 cell-driven inflammation, CTL and NK cell mediated injury of corneal epithelial cells and meibomian glands result in corneal epitheliopathy, corneal ulceration, and meibomian gland dysfunction (MGD) [4,5,6]. The loss of homeostasis, hyperosmolarity of the tears, and persistent intraocular inflammation lead to the neurosensory dysfunction and visual disturbance [4,5,6]. Consequently, dryness, burning, or scratchy sensations in eyes and foreign body sensation are frequently reported by patients with oGVHD [4,5,6]. Severe inflammation may cause punctate epithelial keratopathy and painful epithelial erosions which could be complicated with secondary infections, stromal necrosis, limbal stem cell deficiency (LSCD), and corneal perforation [5]. In addition to DED, uveitis, glaucoma, posterior scleritis, optic nerve edema, retinal detachment, and chorioretinopathy could be also observed in patients suffering from cGVHD and develop due to the detrimental immune response driven by donor T lymphocytes or represent severe complications of immunosuppressive therapy used for the treatment of systemic GVHD [5,6,7].

The three-pronged treatment approach is used as a standard therapy for the treatment of oGVHD [5,6]. Lubrication and tear preservation with topical administration of non-preserved phosphate-free artificial tears is the first-line treatment approach [5,6]. Frequent use of tear substitutes preserves the ocular surface, reduces the concentration of inflammatory mediators at the ocular surface, and prevents aggravation of on-going inflammation [6]. Warm compresses, lid scrubs, and maintenance of lid hygiene should be used as a second-line treatment approach which should prevent tear evaporation and tear film instability [5,6]. Finally, considering the important role of the detrimental immune response in oGVHD development and progression, the attenuation of ocular inflammation represents the third and the most important step in the therapy of oGVHD [5,6]. For this purpose, topical administration of corticosteroids (methylprednisolone, prednisone) and immunosuppressive drugs (Cyclosporin (CsA), Tacrolimus) are used [5,6]. Aggressive topical steroid therapy accompanied with pseudomembrane removal significantly enhanced epithelial healing and suppressed fibrosis in the eyes of oGVHD patients with pseudomembranous conjunctivitis [7]. Topical administration of steroids must not be used for the treatment of oGVHD patients with corneal epithelial defects, stromal thinning, or eye infections [5,6]. Topical CsA and Tacrolimus eye drops were used for the treatment of patients who suffered from mild-to-severe chronic oGVHD with DED refractory to lubrication and steroid therapy [5,6].

In addition to lubrication and immunosuppression, repair and regeneration of injured corneal epithelium and restoration of meibomian and lacrimal glands are also important therapeutic approaches in oGVHD treatment [5]. For this purpose, the administration of autologous serum, which contains epithelial growth factors, cytokines, nerve growth factor (NGF), vitamin A, fibronectin, and TGF-β, was used with partial success [5,6]. The contraindications for the use of autologous serums are the presence of active ocular or systemic inflammation, local or systemic bacterial and viral infections, and poor general health [5,6]. Additionally, due to the strict legal regulations for the use of blood products and transfusions, topical administration of autologous serum eye drops is limited to specialized medical centers [5,6].

Importantly, it should be noted that none of the currently used therapeutic agents was able to suppress on-going eye inflammation efficiently and did not manage to prevent immune cell-driven injury of epithelial cells completely in the eyes of oGVHD patients (Table 1) [7]. The bioavailability of immunoregulatory eye drops is generally low since the well-developed protective mechanisms of the eye ensure their rapid clearance from the pre-corneal space, limiting ocular penetration and therapeutic efficacy of the incorporated drugs [7]. Eye drops which are used in the treatment of oGVHD do not contain growth factors which are able to promote viability and suppress the cell-death-associated signaling pathway in damaged cells. Therefore, none of currently therapeutic agents provided trophic support to the injured cells and did not promote repair and regeneration of injured corneal, meibomia, or lacrimal epithelial cells in the eyes of oGVHD patients [5,6]. Additionally, severe side effects were observed after prolonged use of corticosteroids and immunosuppressive eye drops [7]. Long-term steroid use resulted in the development of glaucoma, cataracts, and corneal thinning in the eyes of oGVHD patients. Similarly, prolonged use of immunosuppressive drugs led to the development of severe immunodeficiency, significantly increasing the risk for the development of secondary infectious keratitis [7]. The existing inadequacies in oGVHD treatment imply that there is an urgent need for the clinical use of new therapeutic agents which will suppress on-going eye inflammation without causing severe immunosuppression and will, at the same time, promote repair and regeneration of injured epithelial cells in the eyes of oGVHD patients.

Mesenchymal stem cells (MSC) are self-renewable adult stem cells which are able to differentiate into corneal epithelial cells under specific culture conditions [8,9]. Additionally, MSC secrete a large number of growth factors that support the viability of injured cells and produce immunomodulatory proteins which regulate the phenotype and function of immune cells that participate in the development and progression of oGVHD [8]. The majority of MSC-derived bioactive factors are contained in MSC-sourced exosomes (MSC-Exos), extracellular vesicles which, due to their nano-sized dimension and lipid envelope, easily by-pass all biological barriers to reach the target epithelial and immune cells in the eyes and lacrimal system of oGVHD patients without affecting neighboring parenchymal cells and, therefore, without causing any severe side effects [8]. Due to their enormous differentiation potential and immunosuppressive characteristics, MSC and MSC-Exos are considered as a potentially new remedy in regenerative ophthalmology [8]. In this review article, we summarized current knowledge about molecular mechanisms which are responsible for the beneficial effects of MSC and MSC-Exos in the therapy of oGVHD.

## 2. MSC-Mediated Tissue Repair and Regeneration: A New Hope for the Treatment of oGVHD

MSC may, under specific culture conditions, differentiate in the cells of all three germ layers [8]. Multi-lineage differentiation potential of MSCs could be a consequence of their complex development origin [8]. During embryogenesis, different subpopulations of MSCs originated from different precursor cells, including epithelial-to-mesenchymal transition-derived cells, Sox1+ neuroepithelial cells, lateral plate mesoderm-derived mesoangioblast cells from the embryonic dorsal aorta, and blood-vessel-derived precursor cells [8].

MSC reside in almost all postnatal tissues from where MSC could be isolated, propagated in vitro, and used in cell-based therapy for degenerative and inflammatory diseases [8]. For clinical use, MSC were the most frequently derived from bone marrow (BM), the umbilical cord (UC), amniotic fluid (AF), and adipose tissue (AT) [8,9]. Main functional properties of BM-derived MSC (BM-MSC), which favor their clinical application, are rapid proliferation in vitro, genomic stability after long-term cultivation, and capacity for the increased production of immunosuppressive cytokines [8]. Although BM-MSC have enormous therapeutic potential, the harvesting of BM is an invasive procedure, and, therefore, UC, AF, and AT were used as alternative tissue sources for the isolation of MSC. The collection of UC-derived MSC (UC-MSC) is a noninvasive, painless, and safe procedure. UC-MSC share similar functional properties with BM-MSC but a have higher capacity for exosome (Exos) production [8]. AF, obtained through amniocentesis, serves as an important source of AF-derived MSC (AF-MSC). AF-MSC produce a large amount of neurotrophins and have high therapeutic potential in the repair and regeneration of injured neural cells [8]. AT-derived MSC (AT-MSC), easily derived from patients’ AT, are usually used for the autologous transplantation of MSC. AT-MSC have a high proliferation capacity and potent immunoregulatory properties [8].

Under specific culture conditions, BM-MSC and AT-MSC may differentiate into corneal epithelial cells [9]. After one week of exposure to the hormonal epidermal medium (SHEM) or standard MSC-cultured Dulbecco’s Modified Eagle Medium (DMEM) supplemented with all-trans-retinoic acid (ATRA), both BM-MSC and AT-MSC managed to differentiate in corneal epithelial cells [9]. A higher expression of epithelial markers (cytokeratin (CK)12, CK3, CK19, E-cadherin) and lower expression of mesenchymal markers (Vim, snail and alpha smooth muscle actin (α-SMA)) were observed in BM-MSC and AT-MSC which were cultured in a SHEM (MSC^SHEM^) or ATRA-supplemented medium (MSC^ATRA^) than in BM-MSC and AT-MSC that grew under standard culture conditions (MSC^DMEM^) [9]. Down-regulation or suppression of the Wnt/β-catenin signaling pathway was crucially responsible for BM-MSC and AT-MSC differentiation toward corneal epithelial cells [9,10]. Importantly, human corneal epithelial cells (HCE) that were co-cultured with MSC^SHEM^ or MSC^ATRA^ had an increased proliferation rate and improved capacity for wound healing than HCE which grew with MSC^DMEM^ [9]. The fact that MSC^SHEM^ or MSC^ATRA^ guided better HCE-driven wound healing than MSC^DMEM^ indicated that SHEM or ATRA not only increased expression of pro-epithelial genes in MSC but also induced enhanced secretion of MSC-derived bioactive factors which improved the viability and proliferation rate of injured HCE [9]. From 720 different proteins which were detected in a BM-MSC and AT-MSC-sourced secretome, 122 proteins participate in the proliferation and differentiation of corneal epithelial cells [8,9]. As suggested by Chen and colleagues, TGF-β receptor type-1, TGF-β receptor type-2, Ras-related C3 botulinum toxin substrate 1, and Ras-related C3 botulinum toxin substrate 2 derived from umbilical cord (UC)-sourced MSC (UC-MSC) were crucially responsible for the MSC-mediated regulation of epithelial cell proliferation [11]. These molecules activate Jun-N-terminal kinase (JNK) and p38 mitogen activated kinase in HCE which elicited signaling pathways that improved their proliferation and migration, crucially contributing to the enhanced healing of corneal wounds [8,9].

In line with these findings are results obtained in several experimental and clinical studies which demonstrated the capacity of MSC to repair and regenerate the injured corneal epithelium, meibomian and lacrimal glands, indicating their therapeutic potential in the treatment of oGVHD [12,13,14,15,16,17,18,19].

By using a rabbit model of alkaline-induced corneal injury, Guo and colleagues showed that human BM-MSC differentiated in corneal epithelial cells and migrated in the damaged corneal stroma where improved survival of corneal stromal cells was observed which finally resulted in corneal regeneration and the attenuation of alkaline-induced corneal damage [14]. In line with these findings are results obtained by Bandeira and coworkers who demonstrated the therapeutic potential of AT-MSC in the treatment of LSCD [15]. Human AT-MSC, which were cultured on fibrin gel and grafted onto the damaged corneal surface of experimental mice, managed to re-populate limbal stem cells and regenerate the injured corneal epithelium [16]. The effectiveness of MSC in LSCD treatment was confirmed in clinical settings as well [17]. BM-MSC successfully engrafted in the eyes of 22 patients suffering from LSCD and significantly improved corneal epithelial failure [17].

By using benzalkonium chloride (BAC)-induced eye injury in rats as an animal model of DED, Beyazyildiz and colleagues demonstrated the therapeutic potential of rat BM-MSC in the regeneration of meibomian glands and in the restoration of meibomian gland function [18]. Reductions in microvilli at apical portions of the corneal epithelium, vascular congestion in meiboian glands, large numbers of apoptotic cells, decreased numbers of goblet cells, a reduced presence of secretory granules, and massive leukocyte infiltration were noticed in the eyes of BAC+saline-treated rats [18]. Topically applied MSC mostly engrafted into the injured meibomian glands, and, in the damaged conjunctival epithelium, MSC suppressed the detrimental immune response and induced the repair and regeneration of injured tissue [18]. Meibomian glands had normal architecture, a significantly increased number of goblet cells with numerous secretory granules, and only a paucity of lymphocytes and neutrophils and a few apoptotic cells were detected in the corneas, conjuctivas, and meibomian glands of BAC+BM-MSC-treated rats. Additionally, the mean aqueous tear volume significantly increased one week after MSC application, suggesting the therapeutic efficacy of BM-MSC in the treatment of MGD and DED [18].

By using a murine model of aqueous-deficient dry eye disease (ADDED), Dietrich and colleagues showed that, in addition to the restoration of the meibomian gland structure, the murine lacrimal gland-derived MSC (LG-MSC) managed to regenerate injured lacrimal glands efficiently as well [19]. ADDED was induced by the ligation of the lacrimal duct. Three days after, duct ligation was removed, and MSC or saline were injected into the lacrimal gland [19]. Duct ligation induced interstitial edema and massive injury of lacrimal glands. Consequently, acinar cells, which produce and secrete the primary tear fluid, were shrunken and dysfunctional in ADDED mice [19]. Immediately after their injection, LG-MSC engrafted in the stroma of lacrimal glands, adjacent to acinar structures [19]. Three weeks after the removal of duct ligation, LG-MSC managed to recover vital acinar structures to 62% of the total lacrimal gland tissue, which is an increase of 25% compared to spontaneous regeneration after saline injection [19]. Tightly arranged acini, organized in lobules and surrounded by connective tissue, were observed in MSC-treated but not in saline-treated lacrimal glands. A higher presence of proliferating, Ki67-positive cells and an enhanced expression of MIST1 expression (acinus specific transcription factor) were observed in the LG-MSC-treated lacrimal glands, confirming LG-MSC-mediated restoration of acinar cells [19]. A significantly reduced expression of caspase-3 in LG-MSC-treated lacrimal glands indicated that LG-MSC suppressed apoptosis of acinar cells [19]. As a result of the LG-MSC-mediated regeneration of lacrimal glands, the amounts of secreted tears in the eyes of MSC-treated ADDED animals were, 21 days after MSC injection, similar to the baseline value which were measured at the ocular surface of healthy animals [19]. MSC-dependent suppression of the detrimental immune response was also responsible for beneficial effects of LG-MSC in the repair and regeneration of lacrimal glands. A significantly reduced number of Ly6G-expressing neutrophils and a lower number of CD68-expressing macrophages were observed in MSC-treated lacrimal glands 21 days after LG-MSC transplantation [19]. Additionally, LG-MSC down-regulated the synthesis of TNF-α in lacrimal gland-infiltrated immune cells and suppressed the TNF-α-driven injury of acinar cells which significantly contributed to the improved tear secretion, suggesting that the immunomodulatory potential of MSC was crucially important for their beneficial effects in oGVHD treatment [19].

## 3. MSC-Dependent Suppression of Detrimental Immune Response in the Eyes as a Potentially New Therapeutic Approach in the Treatment of oGVHD

MSC from all tissue sources are potent immunoregulatory cells that produce large numbers of immunomodulatory factors (IL-10, TGF-β, growth related oncogene (GRO), indoleamine 2,3 dioxygenase (IDO), nitric oxide (NO), interleukin 1 receptor antagonist (IL-1Ra), prostaglandin E2 (PGE2)) which alter the phenotype and function of all immune cells that play a pathogenic role in the development and progression of oGVHD (Table 2) [20].

By suppressing the Jak-Stat signaling pathway in T cells, MSC-sourced TGF-β induces the G1 cell cycle arrest and prevents proliferation of these cells [21]. MSC-derived IDO promotes the expansion of immunosuppressive T regulatory cells (Tregs) and prevents their conversion in inflammatory Th17 lymphocytes [22]. MSC-sourced NO, in an autocrine manner, increases IDO expression in MSC and significantly enhances their immunosuppressive properties [22]. MSC-derived PGE2 attenuates the proliferation of activated T cells and prevents the conversion of naïve CD4+ T cells in effector Th1 and Th17 cells by suppressing IL-2 production in T lymphocytes [21]. Moreover, MSC-sourced PGE2 stimulates the generation of the immunoregulatory tolerogenic phenotype in DC and induces the expansion of alternatively activated macrophages, importantly contributing to the creation of the immunosuppressive microenvironment in inflamed tissues in which MSC were transplanted [22]. Similar to PGE2, MSC-derived IL-10 and TGF-β prevent the generation of inflammatory Th1 and Th17 cells by inhibiting the maturation of DC and by inducing the generation of the alternatively activated (M2) phenotype in macrophages [21,22]. Therefore, the attenuated expression of co-stimulatory molecules (CD80 and CD86) and the suppressed production of pro-Th1 and pro-Th17 cytokines (IL-12, IL-1β, IL-6, IL-23) were observed in MSC-primed DC and macrophages [21,22].

In addition to T cells, DC, and macrophages, MSC are also able to inhibit the proliferation and cytotoxicity of NK cells efficiently [20]. MSC-derived TGF-β and NO suppress the expansion of activated NK cells, while MSC-sourced IDO and PGE2 generate the immunosuppressive and regulatory phenotype in NK cells [21,22]. MSC-derived IL-10 down-regulates the expression of pro-apoptotic and toxic molecules (perforins and granzymes) and inhibits the production of inflammatory and cytotoxic cytokines (TNF-α and IFN-γ) in NK cells, significantly reducing their cytotoxic potential [22].

Juxtacrine communication (the direct cell-to-cell interaction between immune cells and MSC) is also involved in the MSC-dependent suppression of detrimental immunity [20]. MSC express pro-apoptotic molecules (programmed death-ligand (PDL)-1, PDL-2, Fas ligand (FasL)) which bind to PD and Fas receptors on the membranes of activated T and NK cells and induce their apoptosis in a caspase-3-dependent manner [20].

The importance of MSC-based suppression in the therapy of oGVHD was demonstrated by Wang and colleagues who showed that intravenously injected BM-MSC efficiently attenuated T-cell-driven ocular inflammation and alleviated DED in 12 out of 22 BM-MSC-treated oGVHD patients [23]. Clinical symptoms (redness, ocular pain, dryness, scratchiness) were remarkably attenuated in these patients which was manifested with significantly decreased dry eye scores and ocular surface disease index scores [23]. The flow cytometry analysis of immune cells revealed that BM-MSC prevented the activation of cytotoxic CD8+ T cells, as evidenced by a reduced number of CD28-expressing CD8+ T cells [23]. Additionally, BM-MSC alter the cytokine profile of activated CD8+ T cells. A significantly reduced number of pro-inflammatory IFN-γ and IL-2-producing CD8+ T cells and the remarkably increased presence of immunosuppressive IL-10-producing CD8+ T cells were observed in oGVHD patients who received MSC, confirming that MSC-dependent immunoregulation was crucially responsible for their beneficial effects in oGVHD treatment [23]. It has to be highlighted that clinical improvements in DED-related symptoms were noticed in nearly 55% but not in all of BM-MSC-treated GVHD patients which could be explained by the fact that BM-MSC were intravenously infused and were not injected directly in the eyes, so MSC-based immunomodulation relied exclusively on the systemic effects of their secretome [23]. In line with this observation are results of an experimental study conducted by Martinez-Carrasco and colleagues who demonstrated the therapeutic efficacy of subconjunctivally injected human BM-MSC in a murine model of oGVHD [24]. Subconjuctival transplantation of BM-MSC completely attenuated the detrimental immune response and significantly reduced oGVHD in all MSC-treated animals [24]. Massive intraocular infiltration of immune cells, observed in saline-treated animals, was not noticed in the eyes of BM-MSC-treated mice. The total number of inflammatory CD3+ T cells and concentration of inflammatory TNF-α were significantly reduced in the corneas of MSC-treated oGVHD animals [24]. Additionally, subconjunctivally injected BM-MSC suppressed the expression of the PAX6 gene in the corneas of oGHVD mice [24]. Over-expression of the PAX6 gene induces an altered morphology of corneal epithelial cells, increases corneal neovascularization, and promotes the intraocular infiltration of inflammatory immune cells, crucially contributing to the progression of oGVHD [25]. Accordingly, by reducing the expression of the PAX6 gene in the corneas of oGVHD mice, BM-MSC alleviated intraocular inflammation which led to the enhanced regeneration of injured corneal epithelial cells [24].

## 4. Safety Issues Related to the Transplantation of MSC in the Eyes of oGVHD Patients

Due to their potent regenerative and immunoregulatory properties, MSC from all tissue sources were used for the treatment of many incurable degenerative, autoimmune, and inflammatory diseases [20]. In experimental and clinical settings, MSC were injected either directly at the site of injury and inflammation (local transplantation) or were systemically infused (intravenous, intra-arterial, or intra-peritoneal injection) [20]. The majority of locally transplanted MSC successfully engrafted at the site of injury where they (i) secreted growth factors and provided trophic support to injured cells, (ii) produced immunoregulatory factors and suppressed on-going inflammation, (iii) differentiated in parenchymal cells and repopulated damaged tissues. After intravenous injection, the majority of MSC engrafted in the lungs and liver from where, in a paracrine and endocrine manner, through the activity of MSC-sourced immunomodulatory factors, a regulated detrimental immune response was observed [22]. The viability, phenotype, and function of systemically infused MSCs could be altered by cytokines to which they were exposed in systemic circulation and in the tissues of their engraftment [25]. Therefore, significantly better therapeutic effects of MSC were observed after their direct transplantation in the injured/inflamed tissues [20]. In line with these observations, the best therapeutic effects of MSC in the treatment of oGVHD were observed where these cells were topically administered directly in the eyes of oGVHD patients [18].

In addition to MSC, pluripotent stem cells (embryonic stem cells (ESC) and induced pluripotent stem cells (iPSCs)) were also explored as a potentially valuable cell source for the repair and regeneration of injured epithelial cells of oGVHD patients [9,12]. Under specific culture conditions, MSC, ESCs, and iPSCs had a similar potential for differentiation towards corneal epithelial cells [9,12]. Importantly, compared to ESCs and iPSCs, MSC showed a superior potential for the immunoregulation and suppression of the detrimental immune response in the eyes of oGVHD patients [20]. Ethical and safety issues related to the destruction of human embryos, undesired differentiation, and potential malignant transformation limit the clinical application of ESCs and iPSCs [25]. Therefore, among all stem cells, only MSC were considered as potentially novel therapeutic agents for the treatment of oGVHD.

However, it should be noted that there are also safety issues which limit the clinical use of MSC [26,27,28]. Firstly, MSC are not “immune privileged” cells since they express MHC class II molecules [26]. Accordingly, transplantation of allogeneic MSC may aggravate the strong immune response elicited during oGVHD progression [26]. Second, MSC are not exclusively immunosuppressive cells [27]. MSC alter their phenotype and function under the influence of the cytokines to which they are exposed. If MSC are transplanted at the ocular surface or in the vitreous body with low levels of TNF-α and IFN-γ, they obtain a pro-inflammatory phenotype and secrete pro-inflammatory cytokines (TNF-α, IL-1β, IL-6, IL-12, IL-23) which could aggravate Th1 and Th17 cell-driven oGVHD [27]. On the contrary, if MSC are engrafted in the eyes with on-going inflammation (with the high levels of TNF-α and IFN-γ), they acquire an immunosuppressive phenotype and produce IDO, PGE2, IL-10, TGF-β, and other immunoregulatory factors that efficiently attenuate the detrimental immune response [27]. In line with these findings, there is an objective concern that MSC transplanted in the eyes with low concentrations of TNF-α and IFN-γ will obtain a pro-inflammatory phenotype and will aggravate oGVHD [27]. Additionally, TGF-β and bone morphogenetic proteins (BMPs), released by macrophages and parenchymal cells in inflamed eyes, may induce unwanted chondrogenic and osteogenic differentiation of transplanted MSC [28]. Although measurement of inflammatory cytokines and growth factors in the eyes of oGVHD patients prior to MSC injection will minimize the risk for MSC-dependent aggravation of oGVHD, it should be noted that intraocular levels of TNF-α, IFN-γ, and TGF-β are dynamically changing during the progression of oGVHD, and, therefore, concentrations of these cytokines should be continuously monitored in all MSC-treated oGVHD patients [26].

## 5. Therapeutic Potential of MSC-Exos in the Treatment of oGVHD

The majority of MSC-sourced immunoregulatory and growth factors that suppress the detrimental immune response in the eyes and support the regeneration of injured corneas, conjuctivas, meibomian and lacrimal glands are contained within MSC-sourced 50–150 nm large MSC-Exos [29]. As cell-free products, MSC-Exos address all safety concerns related to the transplantation of MSC [26,29]. Furthermore, the lipid bilayer of the MSC-Exos’ membrane enables easy penetration of MSC-Exos through the corneal epithelium and across the blood-retina barrier [26]. Accordingly, topical administration of MSC-Exos has been considered as an alternative therapeutic approach to MSC-based therapy in the treatment of oGVHD [30]. 

MSC-Exos from all tissue sources are enriched with MSC-sourced microRNA (miRNAs) which bind to the RNA-induced silencing complex and inhibit gene expression in target cells [29,30,31]. MSC-Exo-sourced miR-10a-5p and miR-10b-5p prevent apoptosis of injured epithelial cells; miR-191-5p facilitates the cell viability of limbal stem cells, while MSC-derived miR146a suppresses the detrimental immune response by down-regulating the expression of IFN-γ in Th1 lymphocytes [29,30,31]. Labial gland MSC-Exo-sourced miR-125b affected the antibody secretion in plasma cells of patients suffering from DED secondary to Sjogren’s Syndrome by modulating the expression of the PR domain zinc finger protein 1(PRDM1) gene [32]. Accordingly, MSC-Exos significantly reduced the percentage of activated, antibody-producing CD19+CD20-CD27+CD38+ plasma cells in peripheral blood mononuclear cells of these patients and attenuated the antibody-dependent injury of lacrimal glands [32].

AF-MSC-Exos are enriched in neurotrophins (NGF, brain derived growth factor (BDNF)) which provide trophic support to injured neurons and promote axonal regeneration, crucially contributing to the retinal regeneration in the eyes of oGVHD patients [30,31].

AT-MSC-Exos also contain cytokines and growth factors that regulate lymphocyte activation (IL-10, IL-1Ra, TGF-β, GRO, soluble TNF-α receptors (sTNFRs) and promote the repair and regeneration of injured tissues (MMP-2 and 9). In line with these findings are results obtained by Wang and colleagues who demonstrated that MSC-Exos-mediated immunosuppression was mainly responsible for the attenuation of DED in BAC+MSC-Exo-treated mice [33]. AT-MSC-Exos improved the viability of injured epithelial cells by suppressing caspase-1-driven apoptosis [33]. Additionally, AT-MSC-Exos inhibited the activation of the NLR family pyrin domain containing 3 (NLRP3) inflammasome and suppressed the expression of IL-1β and IL-18 in lacrimal-gland-infiltrated macrophages which significantly reduced ocular inflammation and attenuated DED in experimental mice [33].

In line with these findings are results obtained in clinical settings which demonstrated that topical administration of human AF-MSC-Exo-sourced eye drops efficiently attenuated pain, dryness, grittiness, scratchiness, soreness, irritation, burning, watering, and eye fatigue in 131 DED patients [34]. AF-MSC-Exo-sourced eye drops contained IL-1Ra, sTNFRI, sTNFRII, GRO-γ, fatty acid-binding protein 1 (FABP1), and Platelet factor 4 (PF4) which suppressed IL-1β and TNF-α-driven inflammation, prevented the generation of inflammatory Th1 and Th17 cells, supported tear stability, and reduced ocular surface epithelial damage in patients suffering from inflammatory eye diseases [30]. AF-MSC-Exo-sourced eye drops promoted the regeneration of injured meibomian glands and restored meibomian function in patients suffering from MGD [35]. Before the topical application of AF-MSC-Exo-sourced eye drops, meibomian ducts of MGD patients were dilated, while meibomian glands were enlarged and tortuous with an abnormal structure [35]. The morphology of meibomian glands was significantly improved after 21 days of AF-MSC-Exo-based therapy, showing the hypoilluminescent grape-like clusters. Similarly, the hyperilluminescent ducts and underlying tarsus indicated beneficial effects of AF-MSC-Exos in the restoration of the meibomian gland and ducts’ morphology [30]. Significantly improved tear film breakup time was observed 21 days after topical administration of AF-MSC-Exo-sourced eye drops, confirming the restoration of meibomian gland function [30]. Similarly, significantly improved visual acuity, relieved ocular pain, and complete healing of corneal epithelial defects were noticed in AF-MSC-Exo-treated patients who suffered from Sjogren’s Syndrome [30]. In addition to these findings, AF-MSC-Exo-sourced eye drops improved the viability of injured corneal epithelial cells and alleviated symptoms elicited by corneal injury [30]. Four weeks of AF-MSC-Exo-based therapy remarkably improved visual acuity and significantly decreased ocular pain in patients who suffered from epithelial basement membrane dystrophy with recurrent corneal erosion syndrome (RCES) [30]. No recurrence of RCES symptoms was observed in AF-MSC-Exo-treated patients during a follow-up of four months, suggesting beneficial effects of AF-MSC-Exos in the repair and regeneration of injured corneal epithelial cells [30]. Importantly, AF-MSC-Exo-sourced eye drops were well tolerated in all clinical studies, and none of AF-MSC-Exo-treated patients reported any side effects [30,34,35]. 

Only one registered clinical trial (NCT04213248), which is going, to investigate the therapeutic potential of UC-MSC-Exos in the treatment of oGVHD is currently recruiting patients. Patients will receive artificial tears for 2 weeks to normalize the baseline, and, afterwards, UC-MSC-Exo eye drops (10 μg/drop; four times a day) will be administered for 14 days. Changes in the ocular surface disease index, conjunctiva redness scores, tear secretion, tear break time, ocular surface staining, best corrected visual acuity and, tear meniscus height will be determined during the follow-up of 12 weeks. It is expected that the first results of this study will be published in the next two years.

## 6. Therapeutic Potential of MSC-Exos Depends on the Tissue Origin of Their Parental Cells

It should be noted that the content and, therefore, therapeutic potential of MSC-Exos depend on the tissue origin of their parental cells [36]. BM-MSC-Exos are enriched with immunoregulatory factors which induce the generation of the immunosuppressive phenotype in macrophages (TGF-β, IL-10), protect from oxidative stress-induced injury (miR-214), attenuate TNF-α and IL-1β-driven inflammation (sTNFRI, sTNFRII, IL-1Ra), promote expansion of Tregs, and prevent IL-23-dependent generation of Th17 cells (IDO, Kynurenine, TGF-β) [31]. UC-MSC-Exos contain the enzymes manganese superoxide dismutase and glutathione peroxidase 1 which have an anti-apoptotic and anti-oxidation ability and are capable to prevent oxidative-stress-induced injury of neural cells in the eyes of oGVHD patients. Additionally, UC-MSC-Exos may reduce nerve inflammation since they are enriched in proteins which block the degradation and proliferation of the NFκB inhibitor IκBα. UC-MSC-Exos also contain mir-21, miR-23a, miR-125b, and miR-145 which inhibit fibrosis by affecting the factor-β2/SMAD2 pathway and mir-135b-5p, mir-499a-3p which regulate angiogenesis [31]. AF-MSC-Exos contain proteins that modulate neurodevelopment and lymphocyte activation (A disintegrin and a metalloprotease (ADAM)-9, ADAM-10), the repair and regeneration of injured tissues (MMP-2 and 9) and are enriched in proteins that regulate oxidative stress (peroxiredoxin -1,-2,-4,-6). AF-MSC-Exos also contain neurotrophins which provide trophic support to injured neurons in the eyes of oGVHD patients [31]. AT-MSC-Exos have a similar therapeutic potential for oGHVD treatment as other tissues source MSC-Exos. AT-MSC-Exos are also enriched with immunoregulatory proteins IDO, TGF-β, IL-10, IL-1Ra, and PGE2 which suppress Th1 and Th17 cell-driven inflammation and NK-cells-dependent injury of epithelial cells in oGVHD patients [20,31]. The main advantage of AT-MSC-Exos is their high availability since they are easily derived from oGVHD patients’ AT. Accordingly, AT-MSCs may be an alternative when MSC-Exos from other sources have difficulties to extract or are not suitable for therapy [31].

Although MSC-Exos from all tissue sources represent potentially effective therapeutic agents in regenerative ophthalmology, the exact therapeutic dose of MSC-Exos for oGVHD treatment is still unknown [31]. Therefore, upcoming clinical studies should determine the optimal dose, frequency, and treatment schedule before MSC-Exos could be offered as a new remedy for oGVHD treatment. Additionally, the exact growth and immunoregulatory factor(s) which is/are mainly responsible for the beneficial effects of MSC-Exos in the therapy of oGVHD should be defined. Afterwards, MSC could be genetically engineered to over-express these factors which will be contained at high concentrations in MSC-Exos. The administration of MSC-Exos enriched with the most effective bioactive factor(s) will enhance therapeutic potential and efficacy of MSC-Exos in the treatment of oGVHD [31].

## 7. Conclusions

**Figure 2:**
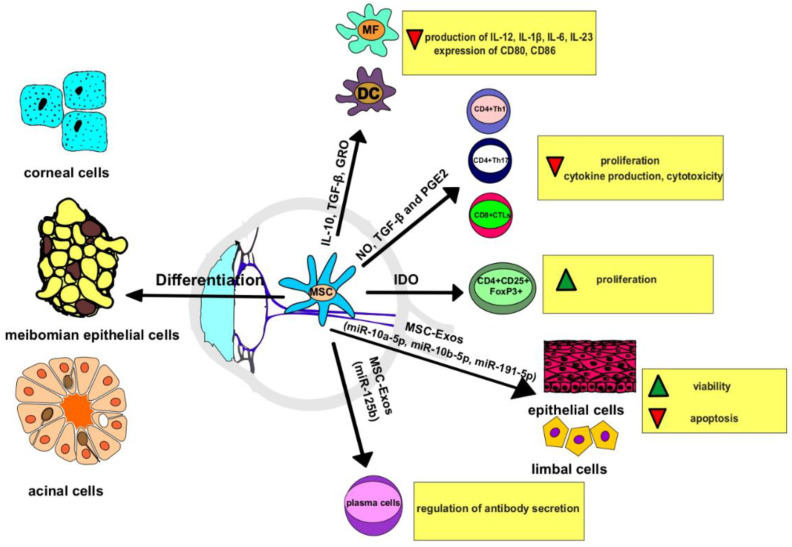
Mechanism of action of MSC and MSC-Exos in oGVHD treatment. MSC differentiate into corneal cells, meibomian epithelial cells, and acinal cells of lacrimal glands. MSC-derived IL-10, TGF-β, GRO attenuate expression of co-stimulatory molecules (CD80 and CD86) and suppressed production of IL-12, IL-1β, IL-6, IL-23 in macrophages and DCs. MSC-derived NO, TGF-β, and PGE2 suppress proliferation and expansion of inflammatory CD4+Th1 and Th17 cells and cytotoxic CD8+ T cells. MSC-derived IDO induces expansion of immunosuppressive Tregs. MSC-Exo-sourced miR-10a-5p, miR-10b-5p, and miR-191-5p prevent apoptosis and facilitate viability of injured epithelial cells and limbal stem cells. MSC-Exo-sourced microRNA-125b regulate antibody secretion in plasma cells in the eyes of oGVHD patients.

Due to their capacity for differentiation in corneal epithelial cells and because of their immunosuppressive properties, MSC enhance the repair and regeneration of the epithelial barrier at the ocular surface, suppress eye inflammation, and restore meibomian and lacrimal glands’ function (Figure 2). Results obtained in experimental and clinical studies demonstrate the therapeutic potential of MSC in oGVHD treatment and suggest that MSC-derived bioactive factors are mainly responsible for their beneficial effects (Table 3) [12,13,14,15,16,17,18,19]. MSC-Exos, which contain all MSC-sourced growth factors and immunoregulatory proteins, due to their nano-size dimension and lipid envelope, easily by-pass all biological barriers in the eyes and deliver their cargo directly in corneal epithelial cells and eye-infiltrated leukocytes [30]. As cell-free agents, MSC-Exos address all safety issues related to the transplantation of their parental cells, including the risk of unwanted differentiation and aggravation of intraocular inflammation [26,31]. Results obtained in experimental and pilot clinical trials demonstrate the therapeutic efficacy of MSC-Exos in the treatment of inflammatory eye diseases and suggest that MSC-Exos could be used in the treatment of oGVHD [30,32,33,34]. Up-coming, large, randomized clinical studies should be conducted to confirm these findings before MSC-Exos could be offered as new therapeutic agents in the therapy of oGVHD.

## Figures and Tables

**Table 1:** The main purpose and weakness of currently used therapeutic agents in the oGVHD treatment and advantages of MSCs/MSC-Exos-based therapy.

Therapeutic Agent	Purpose	Weakness	Advantages of MSCs/MSC-Exos-Based Therapy	Ref. No
artificial tears	lubrication and tear preservation	limited ocular penetration; could not suppress inflammatory immune cells in the eyes; could not enhance viability of epithelial cells	MSC-Exos, due to nano-sized dimension and lipid envelope, can avoid all biological barriers in the eyes and can deliver their cargo directly into the parenchymal and immune cells	[5,8]
corticosteroid-containing eye drops	suppression of eye inflammation	could not promote viability of injured cells; long-term use could result in the development of glaucoma, cataract and corneal thinning	MSCs could differentiate in epithelial cells and may deliver trophic factors in injured cells, enhancing their viability. Long-term use of MSC-Exos could efficiently suppress intraocular inflammation without causing severe side effects	[6,7,8]
immunosuppressive eye drops	inhibition of detrimental immune response in the eyes of oGVHD patients	could not suppress cell-death associated signaling pathway in damaged cells; long-term use could result in the development of secondary immunodeficiency and infectious keratitis	MSCs could enhance viability and may inhibit apoptosis of injured cells in corneal tissue, meibomian and lacrimal glands. Long-term use of locally transplanted MSCs and MSC-Exos could efficiently inhibit eye inflammation without causing secondary infection	[7,8]

**Table 2:** Molecular mechanisms responsible for mesenchymal stem-cell-based immunoregulation in the treatment of oGVHD.

MSC-Sourced Factor	Target Cell	Mechanism of Action	Immunomodulatory Effect	Ref. No
TGF-β	inflammatory Th1 and Th17 cells	inhibition of Jak/Stat signaling pathway; G1 cell cycle arrest	suppressed proliferation of Th1 and Th17 cells	[21]
IDO	T regulatory cells	modulation of T cell receptor signaling	suppressed conversion of T regulatory cells into Th17 cells	[22]
NO	MSC	enhanced IDO activity	expansion of T regulatory cells	[22]
PGE2	naïve T cells	inhibition of IL-2 production	suppressed expansion of activated T cells	[21]
PGE2, TGF-β	DC	inhibition of DC maturation; expansion of tolerogenic DC	down-regulated expression of costimulatory molecules; reduced antigen presentation; decreased production of pro-Th1 and pro-Th17 cytokines; attenuated activation of naïve T cells	[20]
PGE2, TGF-β, IL-10	macrophages	induction of alternative phenotype	increased production of immunosuppressive cytokines	[20,21,22]
TGF-β, NO	NK cells	G1 cell cycle arrest	inhibited proliferation of NK cells	[20,21]
IDO, PGE2	NK cells	induction of regulatory phenotype	expansion of NK regulatory cells	[20,22]
IL-10	NK cells	suppressed production of perforins, granzymes, inflammatory and cytotoxic cytokines	attenuated cytotoxicity of NK cells	[22]

**Table 3:** Experimental and clinical studies that indicated therapeutic potential of MSCs and MSC-Exos in oGVHD treatment.

Animal Models/Patients	MSCs/MSC-Exos	Route of Injection	Mechanisms of Action	Beneficial Effects	Ref. No.
rabbit model of corneal injury	BM-MSCs	intraocular	differentiation in corneal epithelial cells	regeneration of corneal epithelium	[14]
mice model of LSCD	AT-MSCs	intraocular	repopulation of limbal stem cells	regeneration of corneal epithelium	[16]
patients with LSCD	BM-MSCs	intraocular	repopulation of limbal stem cells	regeneration of corneal epithelium	[17]
rat model of DED	BM-MSCs	intraocular	suppression of eye inflammation	regeneration of meibomian glands	[18]
mice model of aqueous-deficient DED	LG-MSCs	intraocular	suppressed apoptosis of acinar cells	restoration of lacrimal glands’ structure	[19]
patients with GVHD associated with DED	BM-MSCs	intravenous	MSC-dependent suppression of CD8+CTLs	reduced injury of epithelial cells	[23]
mice model of oGVHD	BM-MSCs	subconjunctival	MSC-dependent suppressed expression of PAX6 gene in the corneas; inhibition of eye-infiltrated CD3+T cells; down-regulated production of TNF-α in the eyes	attenuated eye inflammation; enhanced regeneration of injured corneal epithelial cells	[24]
mice model of primary Sjögren syndrome	Labial gland-MSC-Exos	intravenous	miR-125b-dependnet modulation of PRDM1 expression in plasma cells	alleviated antibody-dependent injury of epithelial cells	[32]
mice model of DED	AT-MSC-Exos	intraocular	suppression of NLRP3 in macrophages	attenuated eye inflammation; restoration of corneal epithelium	[33]
patients with DED	AF-MSC-Exos	intraocular	Inhibition of eye-infiltrated Th1 and Th17 cells	reduced DED-related symptoms	[35]

## Data Availability

Not applicable.
